# Preclinical study of a Kv11.1 potassium channel activator as antineoplastic approach for breast cancer

**DOI:** 10.18632/oncotarget.22925

**Published:** 2017-12-04

**Authors:** Daniela F. Fukushiro-Lopes, Alexandra D. Hegel, Vidhya Rao, Debra Wyatt, Andrew Baker, Eun-Kyoung Breuer, Clodia Osipo, Jeremiah J. Zartman, Miranda Burnette, Simon Kaja, Dimitrios Kouzoukas, Sarah Burris, W. Keith Jones, Saverio Gentile

**Affiliations:** ^1^ Department of Molecular Pharmacology and Therapeutics, Loyola University Chicago, Stritch School of Medicine, Maywood, IL, USA; ^2^ Department of Pathology, Loyola University Chicago, Stritch School of Medicine, Maywood, IL, USA; ^3^ Department of Chemical and Biomolecular Engineering, University of Notre Dame, Notre Dame, IN, USA; ^4^ Cardiovascular Research Institute, Loyola University Chicago, Maywood, IL, USA; ^5^ Department of Ophthalmology, Loyola University Chicago, Stritch School of Medicine, Maywood, IL, USA; ^6^ Research Service, Edward Hines Jr. VA Hospital, Hines, IL, USA

**Keywords:** cancer therapy, ion channels, activator, DNA damage, senescence

## Abstract

Potassium ion (K^+^) channels have been recently found to play a critical role in cancer biology. Despite that pharmacologic manipulation of ion channels is recognized as an important therapeutic approach, very little is known about the effects of targeting of K^+^ channels in cancer. In this study, we demonstrate that use of the Kv11.1 K^+^ channel activator NS1643 inhibits tumor growth in an *in vivo* model of breast cancer.

Tumors exposed to NS1643 had reduced levels of proliferation markers, high expression levels of senescence markers, increased production of ROS and DNA damage compared to tumors of untreated mice. Importantly, mice treated with NS1643 did not exhibit significant cardiac dysfunction. In conclusion, pharmacological stimulation of Kv11.1 activity produced arrested TNBC-derived tumor growth by generating DNA damage and senescence without significant side effects. We propose that use of Kv11.1 channels activators could be considered as a possible pharmacological strategy against breast tumors.

## INTRODUCTION

Much of the effort directed to targeted anti-cancer drug development has focused on the inhibition of signaling regulators such as receptor tyrosine kinases [1–3]. Such approaches have shown limited effectiveness in treating metastatic disease or in patient subpopulations with cancers that do not express the targeted kinase [4]. In contrast to kinases, ion channels have received relatively little attention as potential cancer therapeutic targets. It is well established that ion channels play major roles in many cellular functions of cancerous cells. Potassium ion (K^+^) channels have been traditionally recognized for their role in controlling important cellular events including electrical transmission, contraction, and secretion. Recent research has revealed that K^+^ channels play fundamental roles in several other important cellular events including cell proliferation [5–7], which is important in cancer.

The human ether-a-go-go-related gene type 1 (hERG1) belongs to the ether a go-go (EAG) gene superfamily initially cloned in *Drosophila* and encodes for the voltage-gated (Kv) K^+^ channel Kv11.1. In adult humans, Kv11 channels are primarily found in cardiac myocytes and in neurons, where they contribute to cell excitability. However, breast cancers of different histogenesis exhibit overexpression of Kv11.1, while the corresponding non-cancerous cells do not exhibit significant levels of the channel. These data suggest that the Kv11.1 channel might be part of a general breast cancer oncogenic signature [8, 9].

Breast cancer is a highly heterogeneous disease that presents differences in morphology and responses to therapeutic options [10]. A fundamental study focusing on molecular characterization of different breast cancers has identified major gene signatures that include cells expressing estrogen receptors (ER+), cells lacking ERs (ER-) and expressing the human epidermal growth factor receptor 2 (HER2+), and cells lacking both ERs and HER2 (triple negative breast cancer; TNBC). Although this discovery has provided the opportunity to develop anti-cancer therapies that selectively aim specific targets and greatly improve the survival of breast cancer patients, particularly the ones bearing ER+ tumors, the same therapies have met limited success in patients with HER2+ and TNBC tumors. Unfortunately, the TNBC tumors also happen to be the most aggressive and present high frequency of recurrence and resistance to treatments [11–13].

In our previous studies, we have shown that pharmacologic stimulation of the Kv11.1 channel current activity with selective and chemically distinct small molecule activators NS1643 or PD115087 induces cell cycle arrest in cultured breast cancer cells that expressed Kv11.1. In contrast, these molecules did not produce any effect in non-transformed breast cells [9, 14, 15]. The antiproliferative effect of Kv11.1 activators coincided with activation of a cellular senescence program in cultured breast cancer cell lines independently of their molecular classification. Senescence is a distinct cellular phenotype that is characterized by permanent arrest of the cell cycle as a response to non-lethal stress such as the formation of reactive oxygen species (ROS) and/or DNA damage [16–20]. Traditionally, cellular senescence can be triggered by telomeric erosion during the normal process of aging or by telomere-independent factors such as oncogenic activation, ionizing radiations (UV or X-rays), and it is generally considered a physiological barrier against tumorigenesis, as senescent cells do not re-enter the cell cycle [21, 22]. Some drugs used in the management of breast cancer, such as the anthracycline doxorubicin, have been found to activate senescence in breast tumors, suggesting that at least in part, senescence mediates the multifactorial cytotoxic effects of doxorubicin on breast cancer cells [23]. Nevertheless, although the ability of doxorubicin to activate senescence appears to provide therapeutic benefit, the occurrence of severe cardiotoxicity [24–28] strongly hampers the use of anthracyclines for treatment of advanced breast cancer.

Several studies suggest an association between ion channels and oxidative stress. For instance, it has been demonstrated that activity of a variety of ion channels, including the Kv11.1 channel, can be directly or indirectly altered by ROS-dependent signaling *in vitro* [29–35] as well as *in vivo* [36, 37]. In several elegant studies, it has been demonstrated that superoxides can produce significant effects on K^+^ channels activity by direct oxidation of specific amino acid residues. Other studies have reported that activity of K^+^ channels can be directly related to the formation of ROS. Further, it is known that Ca^2+^ plays a central role in the process of ROS formation [38]. However, the contribution of ion channels in altering the cancer cell oxidative state is still unexplored. In this study, we aimed to characterize the antineoplastic effects of the Kv11.1 activator NS1643 on TNBC *in vivo* and *in vitro*. The present data demonstrate that NS1643 inhibits TNBC-derived tumor growth *in vivo* in association with increased expression of senescence markers. Acute and chronic application of NS1643 did not produce any significant effect on heart performance.

In addition, we found that NS1643 leads to the production of ROS which results in DNA damage through a Ca^2+^-dependent mechanism. Therefore, we propose that targeting Kv11.1 channels with activators could be considered as a potential pharmacological strategy against breast cancer.

## RESULTS

### Kv11.1 potassium channel gene is overexpressed in human breast carcinomas

**Figure 1:**
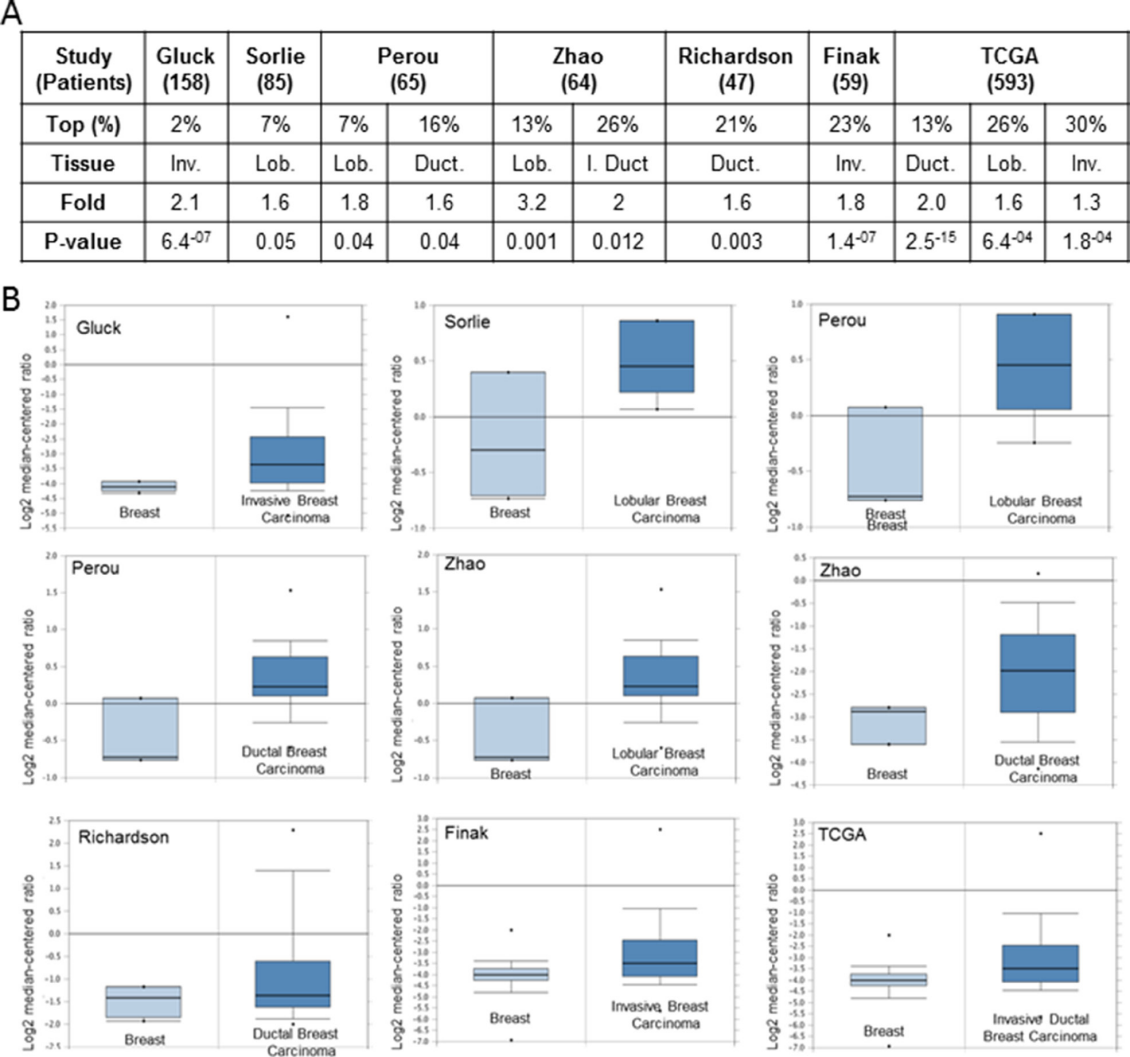
Analysis of KCNH2 mRNA levels in human breast cancer tissues (**A**) Fold = fold change. *P*-value is reported only when < 0.05. Rank = Over-expression gene rank. Type = type of breast cancer: Inv = invasive; I Duct = invasive ductal; Lob = lobular; Duct = ductal.TCGA; http://tcga-data.nci.nih.gov/tcga/). (**B**) Box plot indicating KCNH2 expression level in different studies as indicated. Oncomine dataset; threshold by: P-Value: 1-4; Fold Change: 2; Gene rank: Top 10%.

In this study, we extracted microarray data from the publically available gene expression database Oncomine to examine relative hERG1 (alias KCNH2) gene expression levels in different histological subtypes of breast cancers compared to normal breast tissue and performed biomarker assessment by using Kaplan-Meier Plotter global portal [39]. We found that at least 7 independent studies showed a significant difference in the value of the expression (*p* ≤ 0.05) in breast cancers compared with normal breast tissue (Figure 1A).

In the Gluck’s [40] and Finak’s [41] studies, KCNH2 was significantly upregulated in invasive breast carcinoma and it was ranked among the top 2% and 23% overexpressed genes respectively. While in Zhao’s [42] study, KCNH2 was overexpressed in invasive (top 13%) and invasive ductal (top 26%) vs normal tissue. In Sorlie’s [43] investigation, KCNH2 was upregulated in lobular carcinoma (top 7%) while in the study by Richardson et al. [44], KCNH2 was found to be overexpressed in ductal breast carcinoma (top 21%). Furthermore, in Perou’s study KCNH2 was upregulated in both lobular (top 7%) and ductal (top 16%) breast carcinomas. Finally, in the Cancer Genome Atlas KCNH2 was found to be overexpressed in ductal (top 13%), lobular (top 26%) and invasive (top 30%) breast carcinomas.

### Stimulation of Kv11.1 activity inhibits tumor growth of RasV12 cells

**Figure 2:**
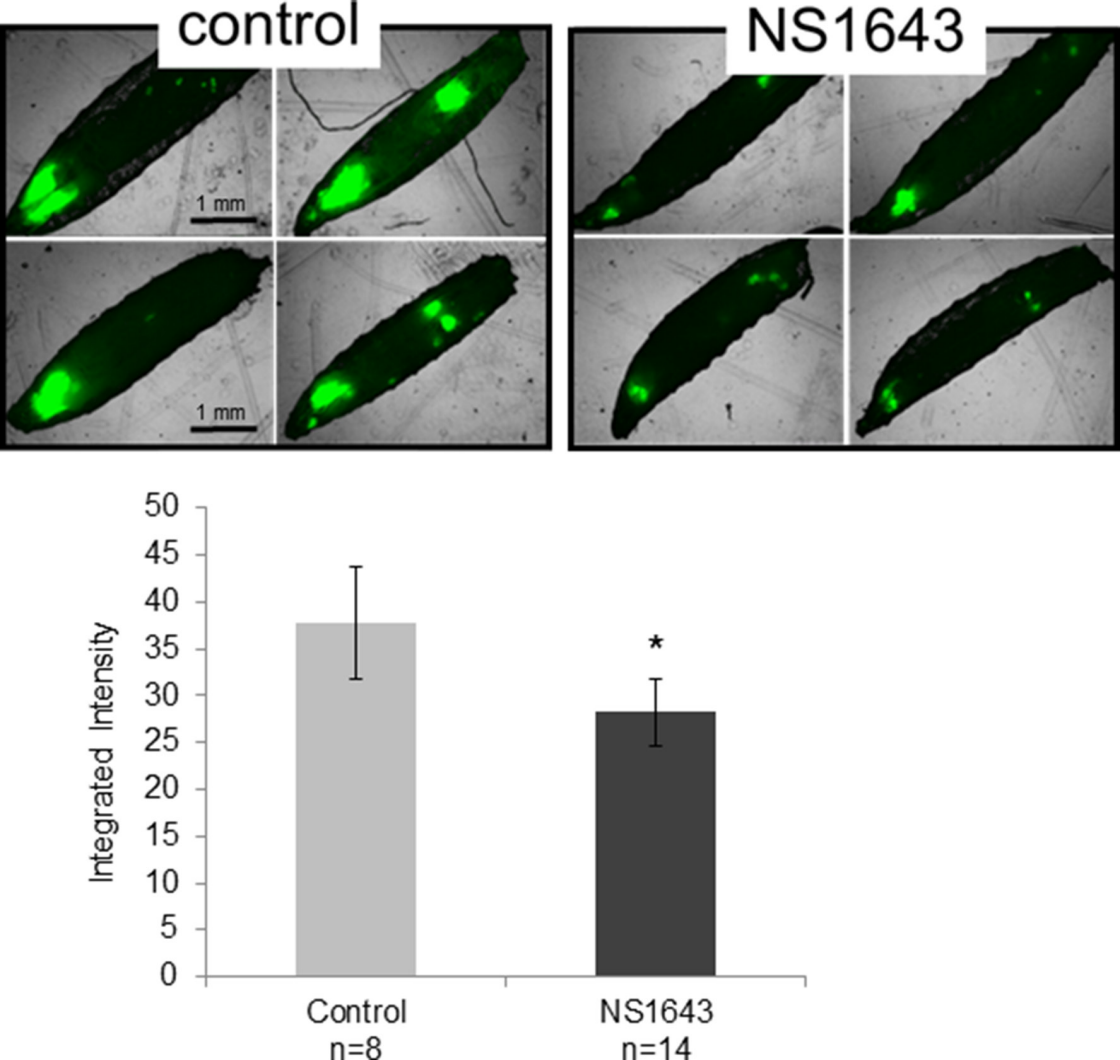
NS1643 arrests RasV12 tumor growth in Drosophila melanogaster Inhibition of proliferation in *Drosophila* model by Kv11.1 stimulation (NS1634) in comparison to vehicle alone (control). On the seventh day after egg laying (3 days of drug treatment), larvae were harvested and imaged on an EVOS fluorescent microscope. Data is expressed as Mean ± SEM; ^*^p < 0.05 (*T*-Test).

The fruit fly *Drosophila melanogaster* is a well-established animal model that has significantly contributed toward elucidating the molecular basis of cancer [45, 46], and more recently has been demonstrated in multiple studies to be an effective preclinical assay for a broad range of cancers in drug discovery [47]. Several recent significant findings have begun to implicate K^+^ channels as important components in morphogenetic signaling [7, 48] *Drosophila* has a Kv11.1/KCNH2 homolog called *seizure* (*sei)* [49–51]. As an initial test in an *in vivo* setting, we investigated the effects of NS1643 on tumor growth in genetically modified *Drosophila* harboring GFP expressing oncogenic RasV12 with knockdown of polarity gene discs large *Dlg*^RNAi^ (*RasV12*, *Dlg*^RNAi^ *tumors)* [52, 53]. *Drosophila* harboring these tumors when fed with Kv11.1 activator NS1643 (25 µM) in their diet developed smaller tumor growth compared to animals that were fed with a vehicle control (Figure 2). These data suggest that application of Kv11.1 channel activator NS1643 can arrest tumor growth and that this response is likely to be strongly conserved across many cancer cell contexts.

### Kv11.1 activator NS1643 inhibits tumor growth in triple negative breast cancer xenograft model of breast cancer

In this study, we wanted to test the efficacy of stimulating the Kv11.1 K^+^ channel activity to in inhibiting TNBC tumor growth *in vivo*. MDA-MB-231 cell line was selected as a TNBC breast cancer cell line, which represents a cancer subtype that is aggressive and has few treatment options. In our previous studies, we showed that stimulation of Kv11.1 activity by the small molecule activator NS1643 inhibits proliferation independently of the molecular characterization of the breast cancer cell line including MDA-MB-231 but no effect of NS1643 was observed in normal cells [2, 11] (Supplementary Figure 1).

**Figure 3:**
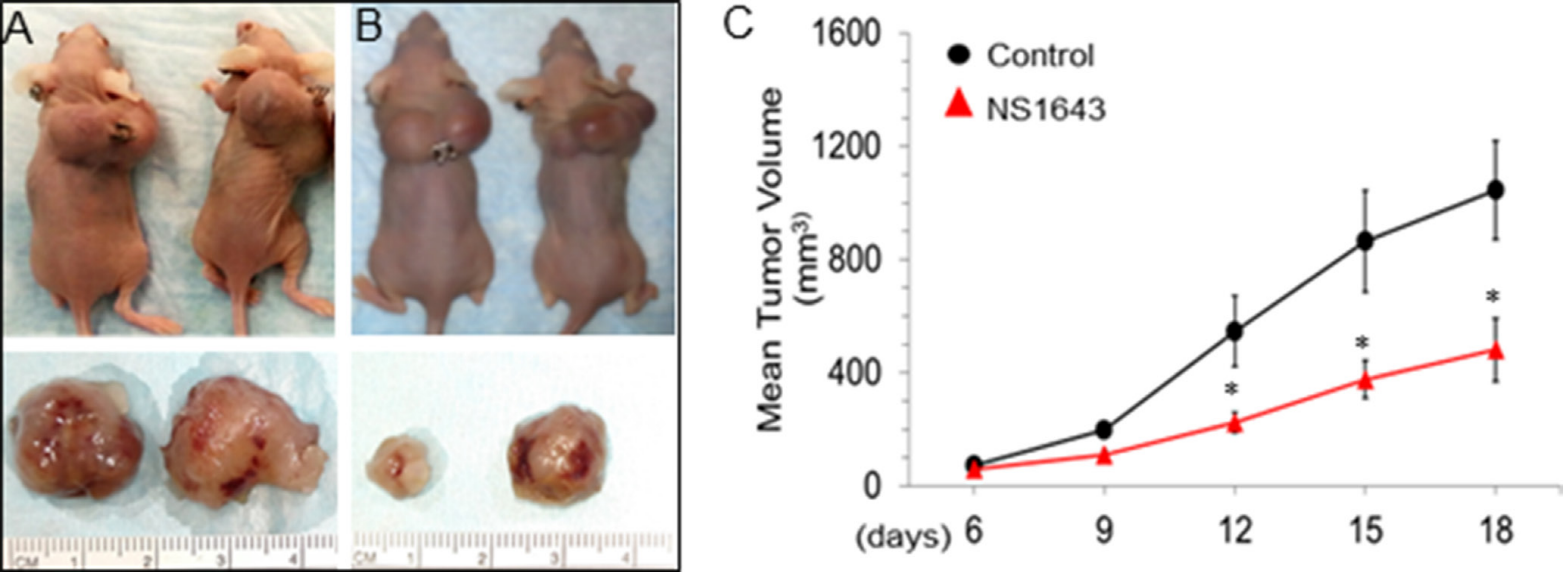
Kv11.1 stimulation inhibits primary tumor growth in xenograft model of breast cancer MDA-MB-231 (ATCC^®^) cells were injected subcutaneously into the flanks of female athymic nude mice. When tumors were palpable the mice were injected intraperitoneally with vehicle alone or Kv11.1 activator NS1643 at 6 mg/kg every two days. (**A**) Representative mice with tumor burdens treated with vehicle (DMSO) alone. (**B**) Representative mice with tumor burdens treated with Kv11.1 activator NS1643. (**C**) Mean tumor volume in mice treated with vehicle alone (control *N* = 6) and in mice treated with Kv11.1 activator (NS1643 *N* = 6). Data is expressed as Mean ± SEM; ^*^p < 0.05.

We, therefore, established a xenograft model of MDA-MB-231 cells in athymic nude mice. In this model, MDA-MB-231 cells developed measurable tumors of 50 mm^3^ as early as 6 days (Figure 3). Mice treated with NS1643 (6 mg/Kg) had significantly reduced tumor volumes in comparison to the vehicle-treated group as early as 6 days following injection (day 12). Smaller dose of NS1643 (1.5 mg/Kg) did not produce significant reduction of tumor growth in the time considered in this study. At the end of the study, we observed that the mean tumor areas of control mice were was1050 ± 200 mm^3^ (Mean ± SEM; Figure 3A, 3C) while that of the NS1643 treated group was significantly lower at 560 ± 120 mm^3^ (Mean ± SEM; Figure 3B, 3C).

### NS1643 suppresses proliferation and promotes senescence in breast tumors

We have shown that NS1643 activates a cellular senescence program [51] in breast cancer cells in culture, a phenotype that is characterized by suppressed proliferation without apoptosis. To confirm that this same mechanism was functional *in vivo* we analyzed the effect of NS1643 in regulating proliferation and senescence markers in tumors in of the MDA-MB-231 xenograft model.

**Figure 4:**
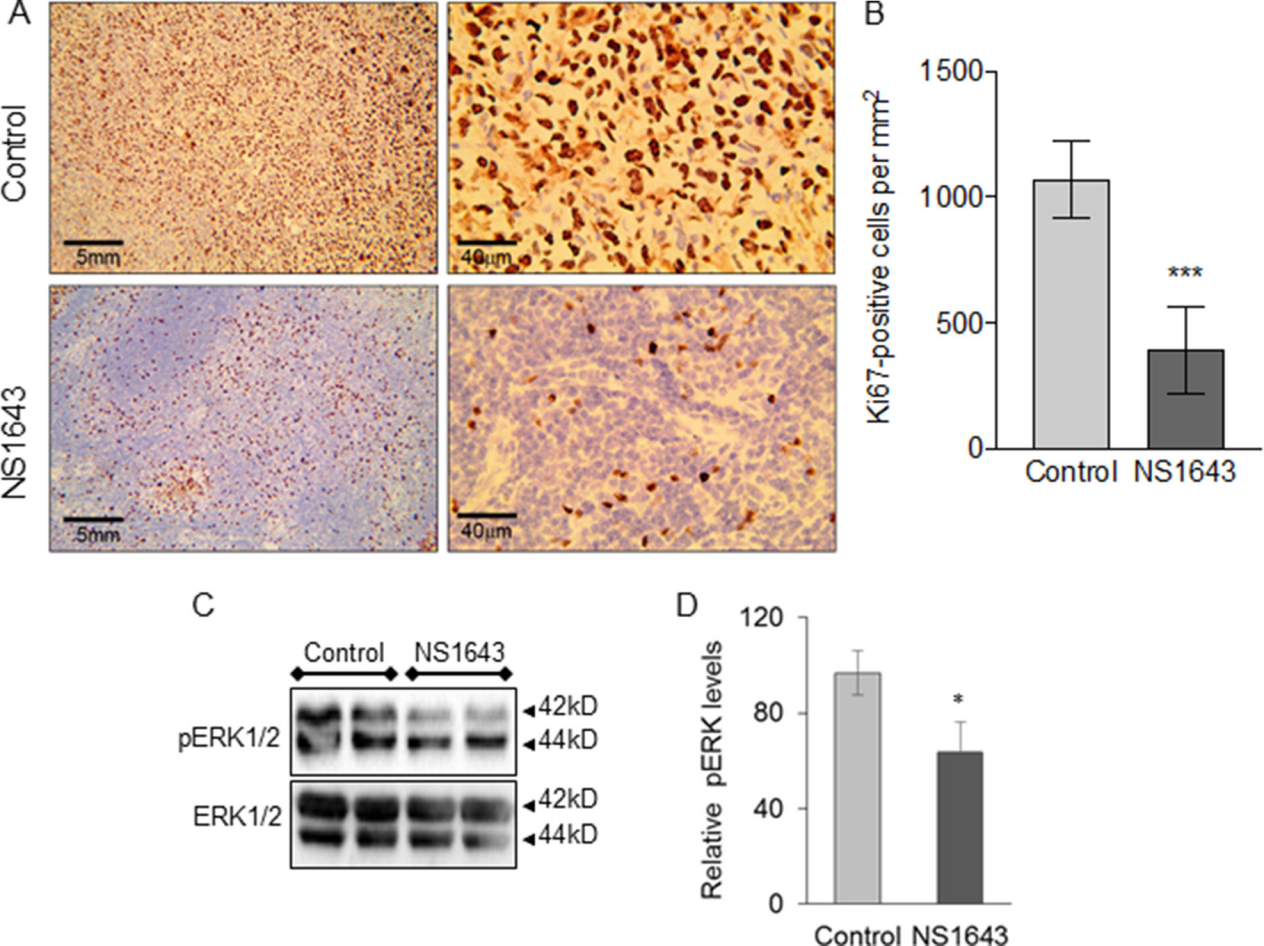
NS1643 treatment decreases tumor proliferation markers (**A**) Detection of Ki67 (clone Mib-1, Glostrup, Denmark) protein expression in mice treated with DMSO (control) or NS1643 and (**B**) quantification of Ki67-positive cells per mm^2^. Data presented as mean ± S.D. Unpaired *t*-test. ^***^p < 0.001; *n* = 5 per group. (**C**) Representative immunoblots demonstrating the effects of NS1643 on phosphorylated ERK or total ERK in tumors extracted from mice receiving DMSO (control) or NS1643. (**D**) Quantification of the effect of NS1643 on activated ERK. Data is expressed as mean ± SEM; Unpaired *t*-test. ^*^p < 0.05 (*n* = 6 per treatment).

High expression of the proliferation marker Ki67 and/or the extracellular signal-related kinase (ERK) levels [52–54] are associated with more aggressive clinical features of TNBC [55–60]. Our immunohistochemistry investigation showed that tumors extracted from mice that received NS1643 presented a significantly lower expression of Ki67 relative to the control group (Figure 4A, 4B). Similarly, we found that the activity of ERK, as indicated by its phosphorylation status, was strongly downregulated in tumors from mice treated with NS1643 compared to control (Figure 4C, 4D).

**Figure 5:**
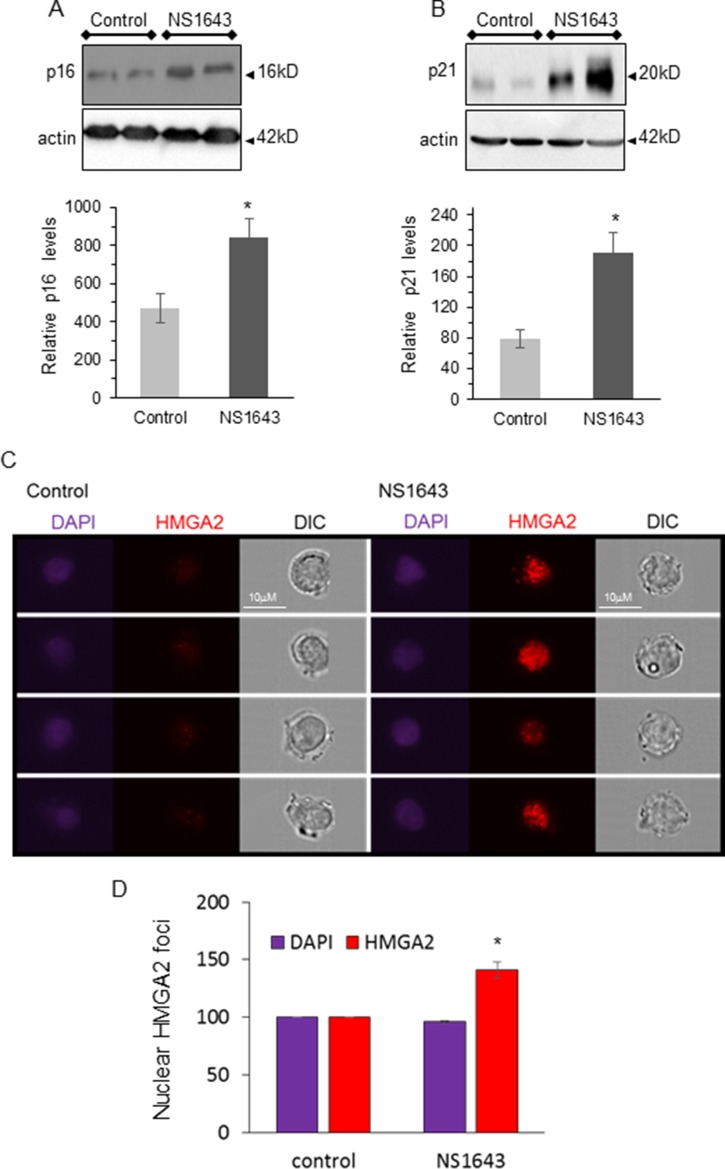
NS1643 treatment increases senescence markers *in vivo* (**A**) Representative immunoblots and quantification of the effects of NS1643 on senescence markers p16^INK4A^ and (**B**) p21^waf/cip^ in tumors extracted from mice receiving DMSO (control) or NS1643. Data is expressed as mean ± SEM; Unpaired *t*-test. ^*^p < 0.05 (*n* = 6 per treatment). (**C**) Representative images of HMGA2 nuclear foci in human breast cancer cells treated with NS1643 or DMSO (Control) for 24 hr. Genomic DNA was stained with DAPI. (**D**) Quantification of nuclear HMGA2 foci in cells treated with NS1643 or (DMSO) control. Shown are 4 representative cells per treatment from one experiment. Two independent experiments were quantified. Data is expressed as mean ± SEM; ^*^p < 0.001.

In contrast, protein levels of the cellular senescence markers p21waf/cip and p16INK4A were increased in tumors from NS1643 treated mice compared to control (Figure 5A, 5B). The high mobility group A2 (HMGA2) is a DNA binding protein non-transcription factor that has been associated with a cellular senescent phenotype [54] and is highly associated with DNA damage [55]. Remarkably, we found that breast cancer cells treated with NS1643 presented a strong increase of the HMGA2 nuclear foci (Figure 5C, 5D), suggesting that HMGA2 can contribute to NS1643-dependent senescence and DNA damage.

### NS1643 generates ROS-dependent DNA damage in breast cancer cells

**Figure 6:**
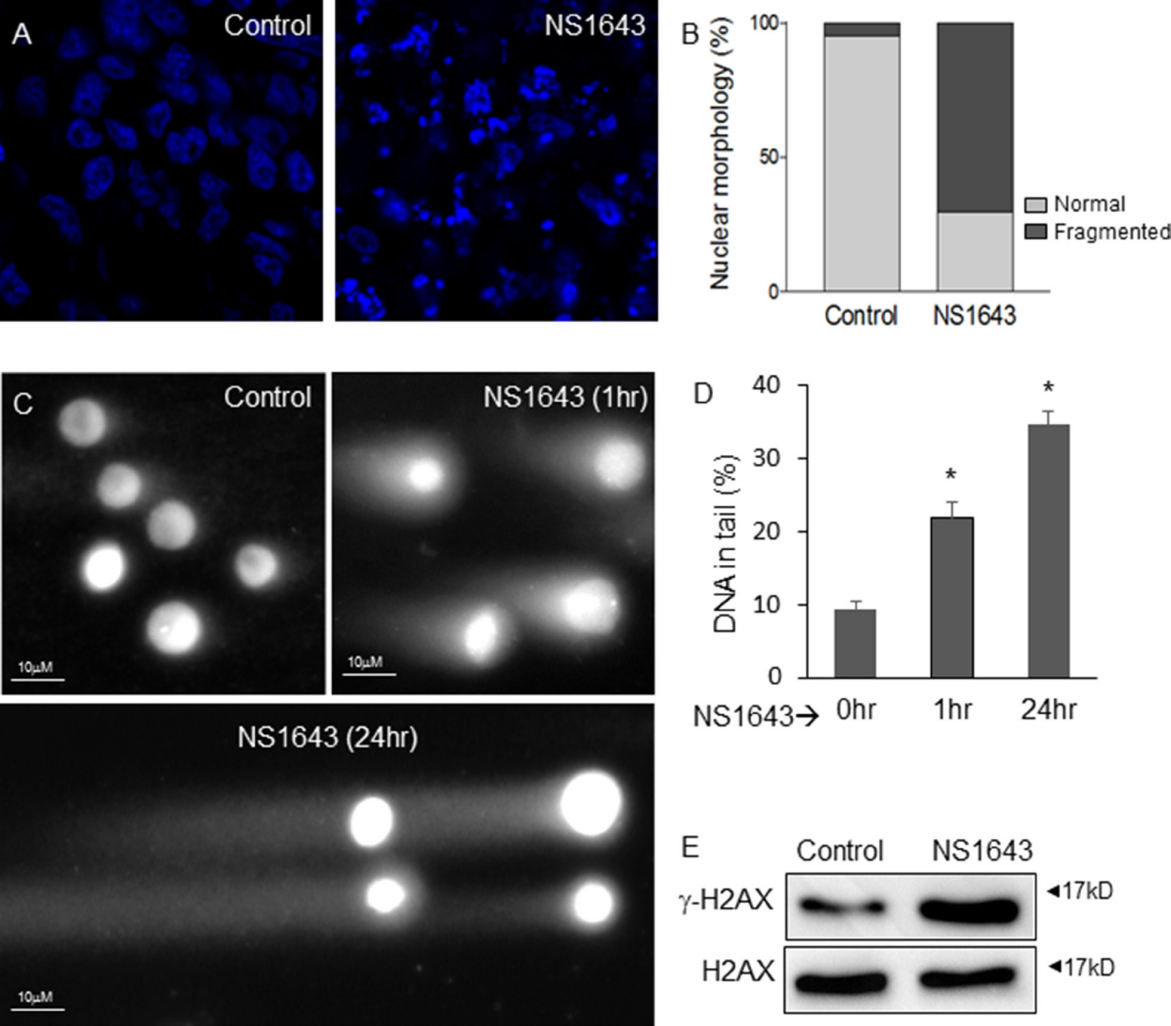
NS1643 generates DNA damage in breast cancer (**A**) Single confocal sections of DAPI-stained tumors from control and NS1643-treated mice show fragmentation of nuclei indicative of DNA damage induced by NS1643 treatment. The number of normal (light grey) vs. fragmented (dark grey) nuclei was quantified by manual counting (*n* = 4, *p* < 0.001, (**B**) Light vs Dark represents normal vs fragmented nuclei. (**C**) Representative images of human MDA-MB-231 cells subjected to neutral comet assay after treatment with NS1643 (50 µM) for 0 hr, 1 hr or 24 hr. (**D**) Quantification of DNA in tail (percent) of cells treated as indicated. Data is expressed as mean ± SEM; Unpaired *t*-test. ^*^p < 0.05 (**E**) Western blot analyses of phosphorylated γH2AX in MDA-MB-231 cells treated with NS1643 (50 µM) for 30 min compared to control (DMSO).

To understand the effect of NS1643 on tumor growth we observed that tumors extracted from NS1643 treated mice exhibited complex DNA fragmentation compared to tumors from untreated mice (Figure 6A, 6B), suggesting that NS1643 could cause DNA damage. Therefore, to better understand the effect of NS1643 on tumor growth, we tested the hypothesis that NS1643 could affect DNA integrity in breast cancer cell lines. A comet assay revealed that application of NS1643 produced significant DNA damage as early as 1h after treatment (Figure 6C, 6D). In addition, NS1643-treated cells showed upregulation of γ-H2AX protein levels, a measure of double-strand DNA damage [56, 57] (Figure 6E) and an increased poly ADP-ribosylation (PARylation), a measure of DNA damage response [58] (Supplementary Figure 2).

**Figure 7:**
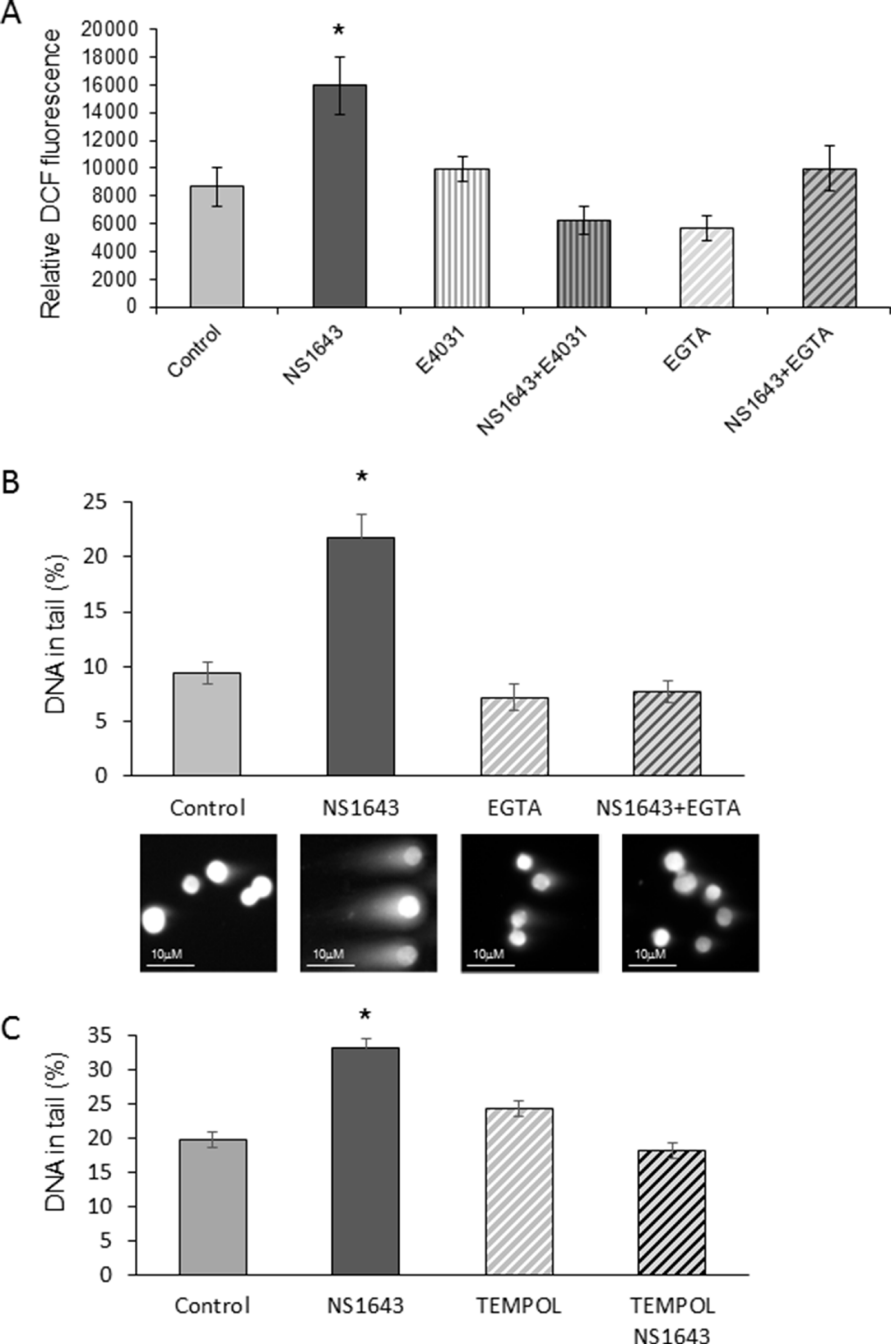
NS1643 generates reactive oxygen species (ROS) and DNA damage in a Ca^2+^-dependent manner (**A**) Effect of NS1643 alone (50 µM), in association with the Kv11.1 blocker E-4031 (10 µM), or in the presence of the Ca^2+^ ion chelator EGTA (2.4 mM) on cellular ROS formation in human MDA-MB-231 cells (DCFH-DA to 2′,7′-dichlorofluorescein DCF, Thermo Fisher Sci; Fluorescence was analyzed in a plate reader (PHERAstar FS, BMG LABTECH) with excitation at 485 nm and emission at 520 nm). Data is expressed as mean ± SEM; ^*^p < 0.001. (**B**) Quantification of DNA in tail (percent) via comet assay of cells treated with NS1643 alone or in the presence of EGTA. Representative images of cells subjected to neutral comet assay are presented below the bars corresponding to specific treatments. (**C**) Effect of the antioxidant TEMPOL (5 mM) on NS1643-dependent DNA damage in MDA-MB-231 cells. Data is expressed as mean ± SEM; ^*^p < 0.05.

As mentioned before, since DNA damage is strongly associated with oxidative stress [6, 59, 60].This led us to we hypothesized that stimulation of Kv11.1 activity could produce ROS formation in breast cancer cells. Results showed that application of NS1643 produced a progressive and significant increase in ROS formation within 2h (Figure 7A) in both MDA-MB-231 (Figure 7A) and in HER2+ breast cancer cell lines (Supplementary Figure 3). Of note, this effect of NS1643 was completely inhibited by the Kv11.1 blocker E4031 (Figure 7A).

In our previous experiments, we have demonstrated that stimulation of Kv11.1 activity correlates with an increase of intracellular Ca^2+^ via passive Ca^2+^ entry [14]. Alteration of cytoplasmic Ca^2+^ can be a major cause of ROS formation [61]. Our experiments showed that generation of both ROS and DNA damage was prevented by treating the cells with the Ca^2+^ ion chelator EGTA (Figure 7A, 7B), suggesting that NS1643-dependent ROS formation and DNA damage were regulated by the Ca^2+^ entry. The same results were obtained in the HER2+ breast cancer cell line SKBR3 (Supplementary Figure 3). In addition, we found that treatment of the MDA-MB-231 cells with the antioxidant TEMPOL rescued the NS1643-dependent DNA damage (Figure 7C). Our data suggest that stimulation of Kv11.1 channel alters the cellular oxidative state which and produces DNA damage.

### Effects of NS1643 on heart function

Cardiac toxicity is one of the most serious adverse effects of anticancer drugs. Cardiac contractility was assessed by transthoracic echocardiograms (TTE) performed on mice with tumors within 1–2h after NS1643 injection [to assess acute effects of the drug] and on mice with tumors that were treated with NS1643 for 25 days (to assess the effect of chronic exposure).

**Figure 8:**
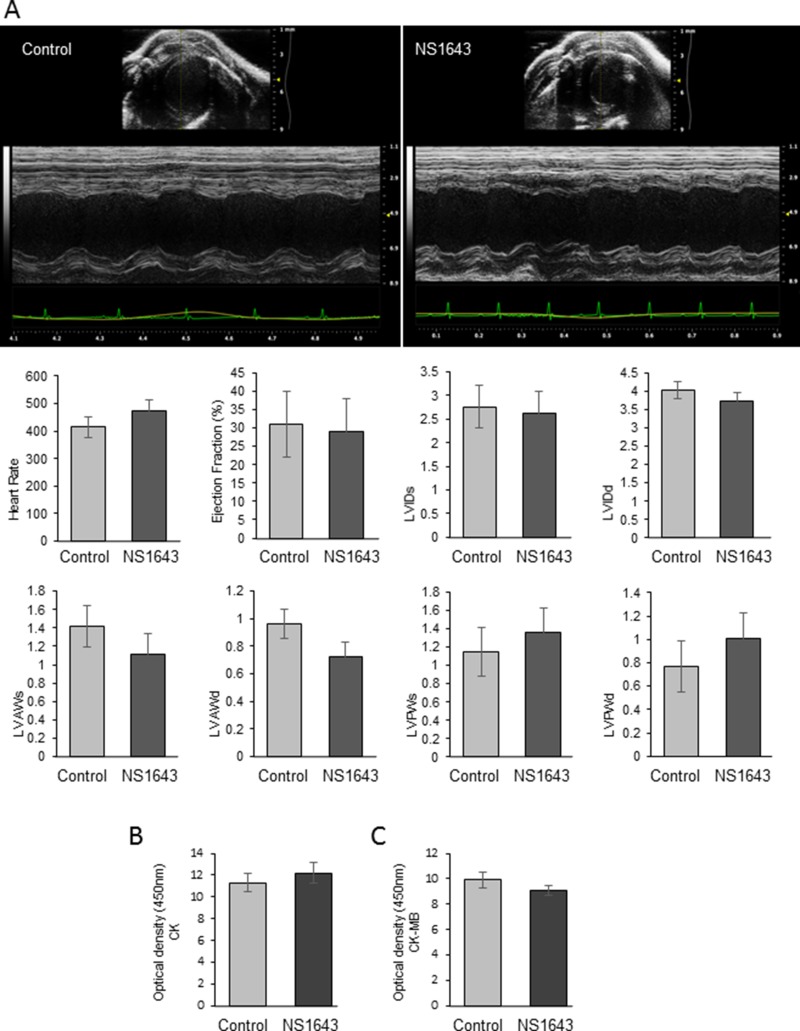
Effect of NS1643 on *in vivo* Cardiac function Transthoracic M-mode echocardiograms echocardiography (Vevo 2100 with MS400 series transducer, Visualsonics Inc.) of (**A**) control (*n* = 3) and NS1643-treated mice (*n* = 3). Figure represents a motion mode of the left ventricle, which is obtained with a single ultrasound beam transmitted through the heart with the resulting image displayed over time. Light Gray bar=control; Dark gray bar=NS1643. HR=heart rate (beats per min); EF=ejection fraction (%); LVIDs & LVIDd=left ventricular internal diameter systole and diastole; LVAWs & LVAWd=left ventricular posterior wall systole and diastole (mm). (**B**) Colorimetric assay detecting serum level of creatine phosphokinase and (**C**) serum level of creatine phosphokinase-Myocardial band (CK-MB) assay in blood extracted from DMSO (control) vs NS1643 treated mice. Beat-to-beat detection was achieved using preset detection and analysis settings for mice (typical QRS width 10 ms, R waves > 60 ms apart, Pre-P baseline 10 ms, Maximum PR 50 ms, Maximum RT 40 ms, ST height at 10 ms, averaging 4 beats). Averaged ECG measurements were taken from a minimum of 20 beats per mouse.

We found that in both acute and chronic exposure, NS1643 did not produce a significant change in any of the measured parameters compared to control (Figure 8; Supplementary Figure 4). To assess whether chronic or acute exposure to NS1643 would generate myocardial damage, we monitored biomarkers for myocardial injuries such as creatine kinase (CK) and CK myocardial band (CK-M) [62]. CK was measured in blood extracted from mice with tumors that were treated with NS1643 and controls for 25 days, while CK-M activity was measured in animals that were treated within 1–2 h before blood extraction. We found that the level of both CK and CK-M activity in blood samples from NS1643 treated animals were not different from controls (Figure 8B, 8C). Overall, these data indicate that exposure to the Kv11.1 activator NS1643 did not significantly alter cardiac function or damage in mice.

## DISCUSSION

The concerted activity of a multitude of ion channels plays a critical role in maintaining homeostasis [7, 63–66] of every living cell by generating and controlling oscillation of ion fluxes across cellular membranes [67–69]. In recent years, the role of ion channels in cancer biology has received significant attention [9, 70]. Particularly, aberrant expression of K^+^ ion channels in cancer tissues, including breast cancers, has been implicated in cancer pathogenesis [71, 72]. It is increasingly recognized that specific changes in ionic concentration across membranes can selectively affect intricate intracellular signaling mechanisms regulating fundamental processes such as cell proliferation, migration, and differentiation [66, 73]. For instance, in our initial study on the effect of NS1643 on breast cancer, we showed that loss of K^+^ ions from the cytoplasm suppresses growth in breast cancer cells as a result of upregulation of tumor suppressors [14, 15, 74, 75]. Accordingly, a recent study demonstrated that accumulation of K^+^ ions in the cytoplasm can stimulate proliferation by amplification of oncogenic signaling such as K-Ras [76]. This indicates that activity of a K^+^ channel such as Kv11.1 can play a critical role in cancer. Although pharmacologic manipulation of K^+^ channels with both activators and blockers strongly affects ionic oscillations and therefore alters cancer cell biology [6, 8, 63, 70, 76–78], still very little is known about the role of K^+^ channels in cancer biology or if they can be considered as potential targets for anticancer therapy.

Our bioinformatics investigation indicates that KCNH2 gene presents a differential expression profile independent of breast cancer histological subtypes, but there is a higher degree of expression in the metastatic phenotype. Although we believe that it is still premature to withdraw a definitive conclusion about the possible role of KCNH2 in oncogenesis, we believe that these data suggest that the KCNH2 gene presents tumor suppressor characteristics at the least in breast cancer. This hypothesis is supported by previous reports showing that the KCNH2 promoter can be found hypermethylated in a variety of cancers, including breast carcinomas, suggesting that in these cancers suppression of Kv11.1 expression might contribute to a hyperproliferative phenotype.

Based on the hypothesis that an increased activity of Kv11.1 can be a limiting factor for cancer cell proliferation, we tested the effects of the small molecule Kv11.1 K^+^ channel activator NS1643 on tumor growth. We demonstrate that NS1643 significantly arrested tumor growth in TNBC MDA-MB-231-induced xenografts and in a Ras-dependent tumor model of Drosophila. Interestingly, none of the mice presented any significant stress signs during the whole duration of the study which might indicate that NS1643 might inhibit the metastatic process in MDA-MB-231. However, our analysis of tissue distribution of metastasis at sacrifice revealed that only 2 control animals presented few and small metastasis (lungs) which indicate the limitation of the current animal model for studying metastatic processes.

Our data provide evidence that chronic stimulation of Kv11.1 produces strong limitation on proliferative potential of breast cancer cells, which overrides oncogenic signals. We found that tumors isolated from NS1643-treated mice presented a strong reduction of proliferation markers, with a concomitant upregulation of cellular senescence markers. Senescence is a form of normal aging resulting from replicative exhaustion and it is generally characterized by a permanent arrest of the cell cycle and loss of cell’s ability to respond to mitotic and/or apoptotic stimuli [17]. We have previously demonstrated that stimulation of Kv11.1 activity produces an increased net negative intracellular electric charge (hyperpolarization) in breast cancer cells [15]. This is important because several studies have demonstrated that a dynamic change in ionic gradients is necessary to promote cell proliferation [66, 79–83]. For example, it has been found that highly proliferative cells present a smaller negative intracellular charge (depolarized) compared to differentiated cells. Therefore, our approach of altering bioelectric controls [84] by “pharmacologically induced forced hyperpolarization” might be functional for inducing senescence and therefore, inhibition of proliferation. In addition, several epidemiological studies have shown that changes in ion intake (e.g. K^+^ or Na^+^) might be associated with carcinogenesis. Although it is not clear what the mechanism linking dietary ions to cancer is, we can speculate that the anticancer effects of targeting ion channels might be potentiated by an appropriate dietary control of slats intake. However, while of obvious etiologic interests, these data should be interpreted with cautions and more dedicated studies need to be developed. Furthermore, cellular senescence has also been detected in benign human tumors [85] and can be activated by several molecular factors, including oncogenes such as RAS, Ca^2+^ or ROS [86]. Interestingly, it has been shown that RAS can alter the cellular oxidation state of the cell, suggesting that ROS plays a critical role in senescence, at least when triggered by an oncogene. Furthermore, ion channels can be directly or indirectly targeted by ROS [29–34], suggesting that these proteins can play a critical role in cellular oxidation processes. Our data demonstrate that stimulation of Kv11.1 activity produced a rapid increase of ROS and a high degree of DNA damage in both *in vivo* and *in vitro*. These data indicate that Kv11.1 K^+^ channel activity controls the production of ROS and it is not only a downstream effector or mediator of oxidative stress.

Furthermore, in our previous work, we have demonstrated that stimulation of Kv11.1 activity can result in a hyperpolarization-dependent Ca^2+^ entry in breast cancer cells [15]. This phenomenon can be explained by the increasing attractive force that hyperpolarization exerts on extracellular Ca^2+^ ions [87, 88]. It has also been shown that Ca^2+^ plays a critical role in the ROS formation process. Remarkably, application of EGTA [a membrane impermeable Ca^2+^ ion chelator] prevented NS1643-dependent ROS formation and DNA damage, suggesting that Kv11.1 controls ROS formation by regulating Ca^2+^ influx.

Overall, our data support the hypothesis that Kv11.1 activity can control Ca^2+^-dependent ROS formation and DNA damage which, contributes to a Kv11.1-induced senescence process. Nevertheless, at this time, we don’t know whether Ca^2+^ entry alone can activate NS1643-dependent ROS production, whether this event requires the intervention of other [e.g. intracellular] Ca^2+^ sources and/or whether mitochondria is necessarily involved in mediating the effects of NS1643. To answer these questions, more mechanistic experiments need to be developed.

Several studies have shown that senescence markers were upregulated in breast cancer samples obtained from patients that were treated with anthracyclines such as doxorubicin. This is an important finding because it suggests that, at least in part, senescence is associated with the antineoplastic effects of doxorubicin and thus finding drugs that can trigger senescence could have a significant impact on cancer therapy. Unfortunately, cardiac toxicity associated with these drugs can result in the irreversible and lethal condition of congestive heart failure and it is often the cause for discontinuing the treatment. Cardiac toxicity is also an important limiting factor for potential therapeutic molecules that act on Kv11.1. It is well known that Kv11.1 blockers can be associated with prolongation of the QT (Long QT; LQT) interval in the heart’s electrical cycle which is a marker for ventricular fibrillation and a risk factor for sudden death [89–91]. For example, several studies have routinely tested the effects of Kv11.1 inhibitors on different cancer cells but, despite their success in impeding cancer cell proliferation, use of these Kv11.1 blockers as anticancer therapeutics is discouraged due to the associated, often lethal severe cardiac arrhythmias [73, 77, 78, 92, 93]. In contrast, cardiac side effects related to Kv11.1 activators (which were initially tested to correct LQT) might include tachycardia (increased heart rate) [94].

Remarkably, our echocardiogram experiments demonstrate that mice treated with acute application of the Kv11.1 activator NS1643 did not present significant alteration of cardiac performance or changes in biochemical parameters that are typically associated with drugs acting on Kv11.1 channel. Our experiments revealed a slight increase in the heart rate in mice treated with NS1643 compared to untreated mice; however, these changes were not statistically significant. In addition, no alteration of the LQT was observed in NS1643-treated mice. Nevertheless, studies on the effect of NS1643 or other Kv11.1 channel activators in humans are still lacking. However, at this time we can speculate that eventual acute effects produced by these drugs on heart performance could be corrected by Kv11.1 blockers which are routinely used in clinic. In addition, chronic exposure to NS1643 did not reveal any significant side effects on the heart caused by current chemotherapeutic or targeting agents (e.g. doxorubicin). Therefore, our data suggest that at the effective dose for arresting tumor growth, NS1643 does not elicit severe cardiac side effects. We would like to point out that tachycardia is easily corrected by the routine measure of intervention [e.g. by beta-blockers] during treatment of patients with breast cancer. Therefore, in comparison to the severe often fatal side effect associated with the use of current antimetastatic agents [24, 25] [e.g. doxorubicin-dependent congestive heart failure], the potential benefits of Kv11.1 activators as anticancer drugs outweigh their potential side effects.

We have previously shown that while chemically distinct, Kv11.1 activators arrest proliferation in breast cancer cell lines without producing any effects on normal cells. Therefore, our experiments are significant and important because they indicate that pharmacologic stimulation of Kv11.1 can selectively force breast cancer cells to undergo cellular senescence and arrest tumor growth without causing major cardiac toxicity.

Although much more research is needed to understand the full benefit of targeting ion channels as an anticancer therapy, we propose that stimulation of the activity of K^+^ channels such as Kv11.1 could be considered as a potential approach to design a novel anti-breast cancer therapy. Overall, these studies demonstrate that manipulation of ion channels activity could be considered as an important and novel tool to better understand cancer biology and to develop potential anticancer approaches.

## MATERIALS AND METHODS

### Cells, antibodies, and reagents

All cell lines were purchased from ATCC and maintained at 37°C and 5% CO_2_ in Dulbecco’s modified Eagle’s medium (DMEM, 4.5 g/L glucose), supplemented with 10% fetal bovine serum, non-essential amino acids, 1% Penicillin (100 mg/ml) and streptomycin (100 mg/mL) antibiotics. Mouse anti-Ki-67, rabbit anti-phospho-ERK1/2, ERK1/2, p21^waf/cip^, and HMGA2 antibodies were purchased from Cell Signaling Technologies, Inc (Boston, MA, USA). Mouse anti-p16^INK4A^ antibody was purchased from Sigma (St. Louis, MO, USA), Mouse anti-phospho H2AX antibody was purchased from EMD Millipore (Darmstadt, Germany), and rabbit anti-H2AX antibody was purchased from Abcam (Cambridge, MA, USA). Horse anti-mouse and goat anti-rabbit secondary antibodies were purchased from Cell Signaling Technologies, Inc. (Boston, MA, USA). NS1643 was purchased from Alomone Labs (Jerusalem, Israel), EGTA was purchased from Calbiochem (San Diego, CA, USA), TEMPOL was purchased from Sigma (St. Louis, MO, USA) and E-4031 was purchased from Abcam (Cambridge, MA, USA).

### Drosophila tumor metastasis

The “tumor tester” line with genotype eyFLP; UAS-RasV12, UAS-dlgRNai, UAS-GFP/CyO, GAL80ts [44] was outcrossed to yw to eliminate Gal80ts, which inhibits GAL4 driven transgene expression. After four days of tumor progression at 25°C, GFP-positive [tumor-bearing] larvae were harvested and relocated to new vials with Drosophila food treated with either: NS1643 (25 µM, in DMSO) or vehicle control (DMSO). Three days after drug treatment, larvae were harvested and imaged on an EVOS fluorescent microscope. Data are expressed as Mean±SEM of the integrated GFP intensity in the larvae; ^*^p < 0.05, one-tail *t*-test.

### Tumor xenograft models

Female athymic nude mice (*n* = 12), aged 6 weeks, received a subcutaneous injection of 4 × 10^6^ MDA-MB-231 cells into the flanks – (100 mL 1:1 PBS). Mice were randomly divided into two groups (*n* = 6): control (A) and NS1643 treatment (B) Mice in the NS1643 treatment group received NS1643 (6 mg/Kg/2days interval) while the control group received DMSO by intraperitoneal (IP) injections. Tumor size was measured every 3 days using a digital caliper. All animal study protocols were approved by Loyola University’s Institutional Animal Care and Use Committee

### Western blot analysis

Tumors were grinded with a pestle and lysed with a buffer containing 50 mM HEPES, 1% Triton X-100, 150 mM NaCl, 5 mM EDTA, 1mM Na3VO4, 1 mM NaF, 1 mM PMSF, and 1x Protease inhibitor cocktail (Cell Signaling Technologies Inc, Boston, MA, USA). Cultured cells were harvested by trypsinization with 0.25% Trypsin-EDTA, washed in PBS, and lysed with cold radioimmunoprecipitation assay (RIPA) buffer (50 mM Tris HCl (pH 8.0), 150 mM NaCl, 1% Tergitol (Sigma-Aldrich, St. Louis, MO, USA), 0.5% Na-deoxycholate, 0.1% SDS, 1 mM PMSF, 1 mM NaF, 1 mM Na3VO4, and 1 × Protease Inhibitor Cocktail (Cell Signaling Technologies Inc, Boston, MA, USA). Protein concentration was determined by BCA assay (Thermo Scientific). An equal amount of protein samples were incubated with 2X or 4X Laemmli buffer at 95°C for 5min. The samples (32 μg) were subjected to SDS-polyacrylamide gel electrophoresis on 4–15% gradient mini-gels and transferred onto a nitrocellulose membrane. Membranes were blocked with 5% nonfat milk in Tris-Buffered Saline containing 0.1% Tween 20 (Sigma-Aldrich) (TBST). Following washes with TBST, the membranes were incubated overnight at 4°C with primary antibodies diluted 1:1000 in 5% bovine serum albumin in TBST. The membranes were then washed with TBST and incubated with anti-rabbit or anti-mouse secondary antibody (1:2000) in TBST with 5% nonfat milk for 1h at room temperature. Following secondary antibody incubation, the membranes were thoroughly washed with TBST and visualized using Luminata Forte Western HRP Substrate [EMD Millipore, Darmstadt, Germany].

### Immunohistochemistry of Ki67 protein expression in xenograft breast tumors

Paraffin sections of four tumors from mice that received DMSO or NS1643 were incubated with the Ki-67 antibody at a dilution of 1:200. A polyclonal biotinylated F’ab antibody to mouse immunoglobulin G reactive with all mouse isotypes and a streptavidin–biotinylated peroxidase complex or the Envision kit (Dako, Carpinteria, CA, USA) served as detection systems for the primary antibody. The sections were faintly counterstained with Harris’ hematoxylin. Negative controls were performed without primary antibody.

### HMGA2 foci

Human MDA-MB-231 cells were treated with NS1643 or DMSO (control) and stained with DAPI (purple) and immunostained with an antibody recognizing HMGA2 (red). For each sample, a minimum of 75 cells was analyzed using LSM710 ZEN software and the mean percentage value+SE was determined.

### PAR immunocytochemistry

Cells plated on coverslips (*N* = 2/group) were fixed (4% PFA in PBS, 20 min), and blocked (10% normal goat serum (NGS), 1% BSA, 0.5% TritonX-100 in PBS, for 1 hr). Coverslips were labeled (2 hrs) with an anti-PAR mouse monoclonal antibody (1:100; ALX-804-220; Enzo Life Sciences, Farmingdale, NY) in antibody diluent (3% NGS, 1% BSA, 0.5% TritonX-100). After PBS washes (3 × 5 min), coverslips were incubated (1 hr) with an Alexa 594-conjugated secondary antibody (1:1000; 115-585-166; Jackson ImmunoResearch Laboratories, West Grove, PA) in antibody diluent (3% NGS, 1% BSA, 0.5% TritonX-100). Coverslips were incubated with DAPI (1:11 in PBS; NucBlue; Molecular Probes, Eugene, OR; 10 min) and washed (PBS, 3 x 5 min), before mounting on microscope slides (Aqua-Poly/Mount; Polysciences, Inc, Warrington, PA) and drying. All procedures took place at room temperature.

Images were collected in DAPI and Alexa 594 channels using an inverted wide-field fluorescence microscope (IX81, Olympus, Center Valley, PA) equipped with a 12-bit CCD camera (QIClick; QImaging, Surrey, BC Canada) and a motorized stage (Proscan III; Prior, Rockland, MA). Constrained iterative deconvolution was performed on image stacks using cellSens software (Olympus, Center Valley, PA). Regions of interest (ROIs) around nuclei were created by thresholding DAPI images in ImageJ (NIH, Bethesda, MD), and PAR immunofluorescence within ROIs was then quantified.

### DNA damage detection

Tumors from mice that received DMSO or NS1643 were excised and processed for cryosectioning. 12 mm thick sections were stained with DAPI (100 ng/µL) and imaged using a Leica SPE confocal microscope (Leica Microsystems). The number of nuclei exhibiting fragmented vs. normal morphology was counted manually in ImageJ software. Data are expressed as a fraction of the normalized total number of nuclei.

Neutral comet assay was performed for detecting DNA double-strand breaks in human MDA-MB-231 cells. The images were captured on a fluorescent microscope and quantified by using TriTek CometScore ™

### Detection of reactive oxygen species (ROS)

Cells were seeded at 4 × 10^4^ cells/ml in 96 well plates. Adhered cells were incubated in the dark with DCFH-DA to 2′,7′-dichlorofluorescein (1 h/37°C; 40 μM, ThermoFisher Sci). Cells were washed and subjected to different drug treatments for 2h. Fluorescence was analyzed in a plate reader (PHERAstar FS, BMG LABTECH) with excitation at 485 nm and emission at 520 nm

### Echocardiogram and ECG analysis

Cardiac function was assessed using echocardiography (Vevo 2100 with MS400 series transducer, Visualsonics Inc.). Briefly, mice were anesthetized with 1.2-1.5% isoflurane and placed on a heating pad to maintain body temperature. Parasternal short-axis 2-D echocardiograms and M-mode cine loops were taken at the level of the papillary muscles. The total length of anesthesia was less than 10 mins. Evaluation of stored data was performed offline by a sonographer blind to treatment groups.

### ECG analysis was performed in LabChart8 (ADInstruments)

#### CKs activity

Creatine kinase (Abcam) and myocardial creatine kinase (MyBioSource) activity were measured by ELISA method in blood samples extracted from NS1643-treated or untreated mice according to manufacturer instructions.

## SUPPLEMENTARY MATERIALS FIGURES



## Abbreviations

K^+^: potassium
Ca^2+^: calcium
HERG: human ether-a-go-go-related gene type 1
Kv: voltage-gated potassium channel
ER+: estrogen receptor
HER2: human epidermal growth factor receptor 2
TNBC: triple negative breast cancer
ROS: reactive oxygen species
HMGA2: high mobility group A2
EGTA: ethylene glycol-bis(β-aminoethyl ether)-N,N,N’,N’-tetraacetic acid
TTE: transthoracic echocardiograms
CK: creatine kinase
CK-M: creatine kinase-myocardial band
