# Supplementary material for: Preclinical study of a Kv11.1 potassium channel activator as antineoplastic approach for breast cancer
Source: Oncotarget. 2017 Dec 4;9(3):3321–37. doi: 10.18632/oncotarget.22925 (PMC5790466; doi:10.18632/oncotarget.22925)
Supplement: Supplementary file 1 [file oncotarget-09-3321-s001.pdf]

Preclinical study of a Kv11.1 potassium channel activator as antineoplastic approach for breast cancer

SUPPLEMENTARY MATERIALS

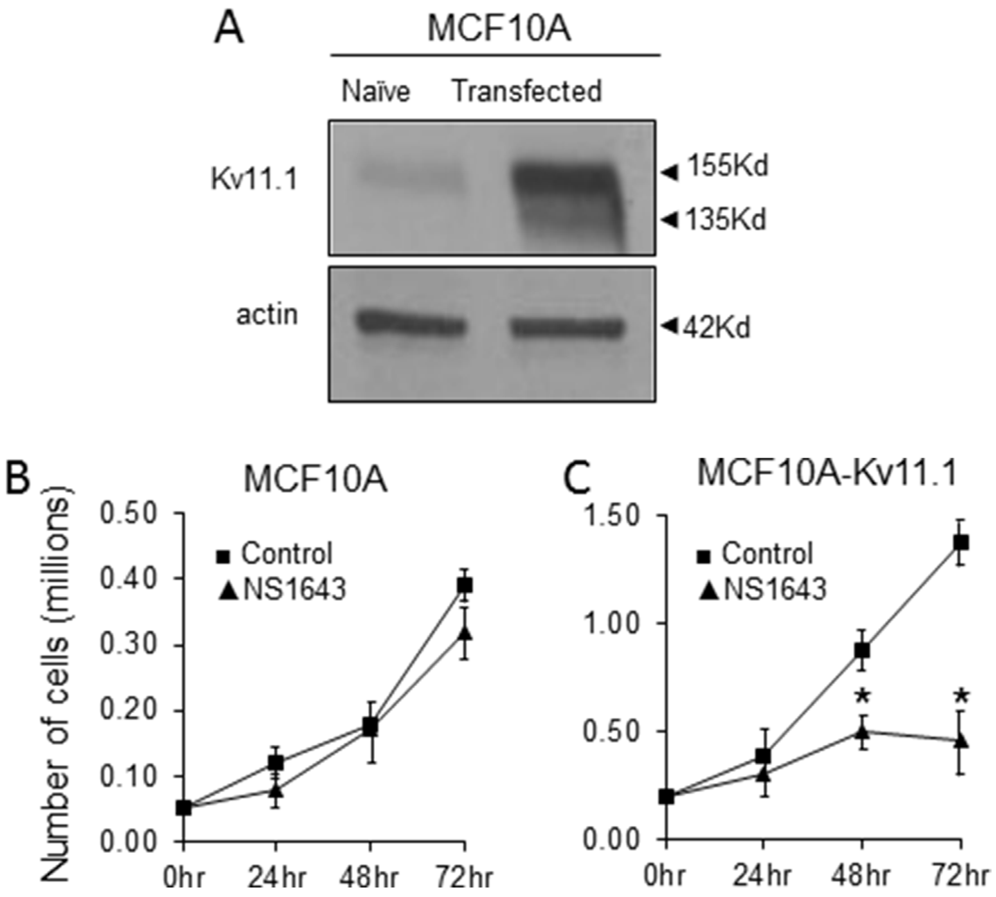

Supplementary Figure 1: Effect of NS1643 on non-transformed cell exogenously expressing Kv11.1. (**A**) Western blot from MCF10A expressing exogenous Kv11.1 (MCF10A-Kv11.1) compared to naïve cells. (**B**) Effect of NS1643 on proliferation rate of naïve MCF10A or (**C**) MCF10A-Kv11.1.

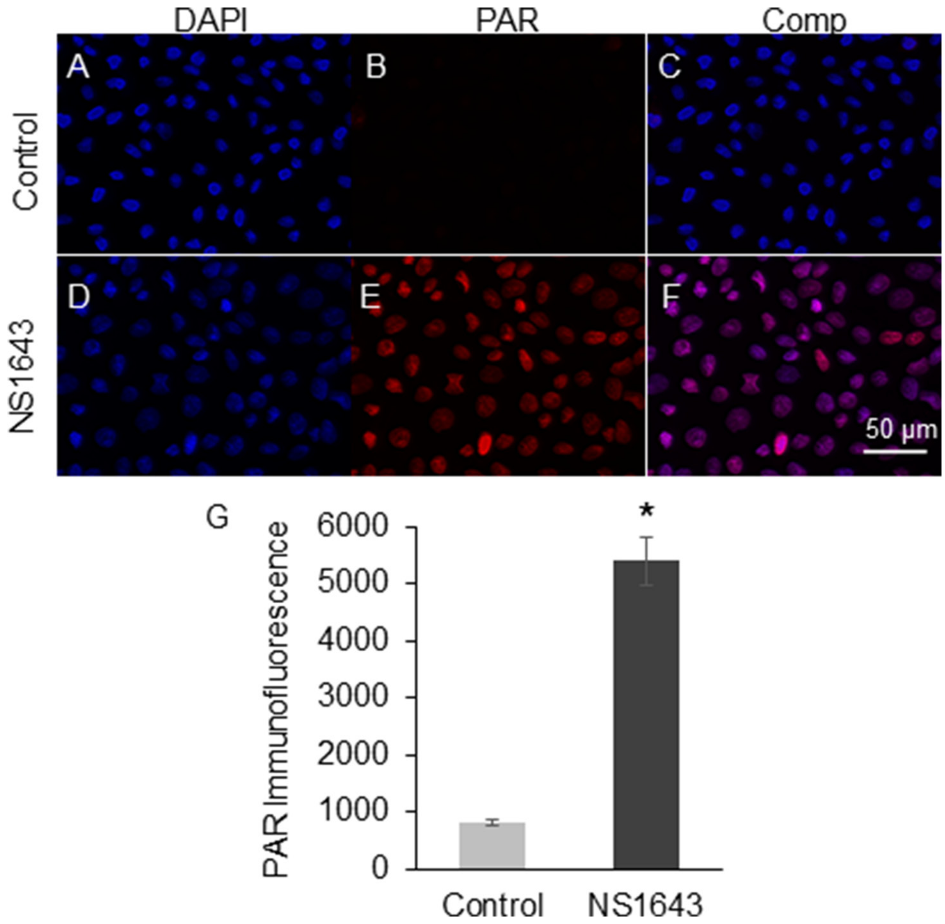

Supplementary Figure 2: PARylation increases in NS1643-treated breast cancer cells. (**A–D**) shows nuclei stained with DAPI (A and D) and PAR immunofluorescence (**B** and **E**) in MDA-MB-231 cells treated as indicated. Composite images (**C, F**) indicate that NS1643-induced PARylation localized to cell nuclei. Analysis of PAR immunofluorescence (**G**) revealed that NS1643 elevated nuclear PAR by 12.6 times $^{*}p < 0.05$.

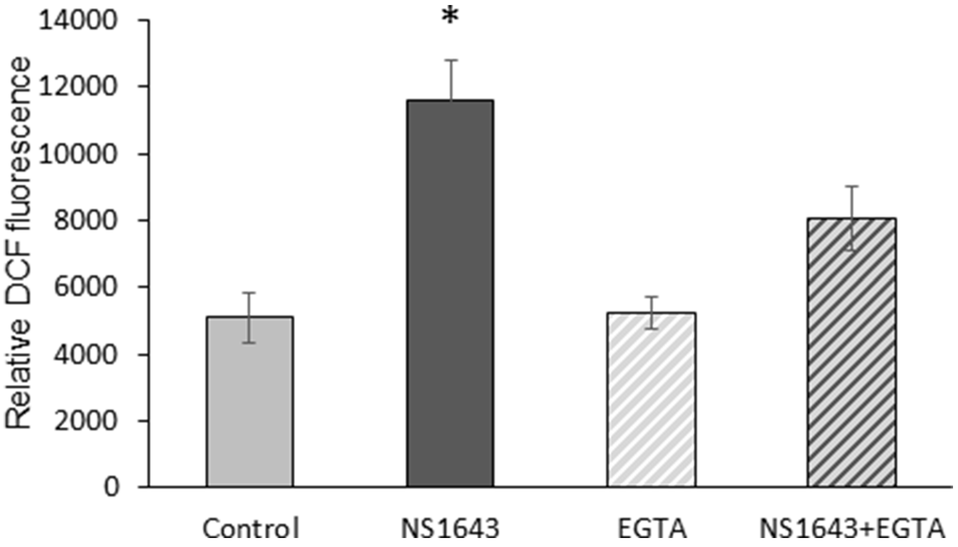

Supplementary Figure 3: NS1643 generates reactive oxygen species (ROS) and DNA damage in a Ca^{2+}-dependent manner in HER2+/ER- SKBr3 cells. Effect of NS1643 alone or in the presence of the Ca^{2+} ion chelator EGTA on cellular ROS formation (**A**) and DNA damage (**B**) in SKBR3 cells. Data is expressed as mean ± SEM; $^{*}p < 0.001$.

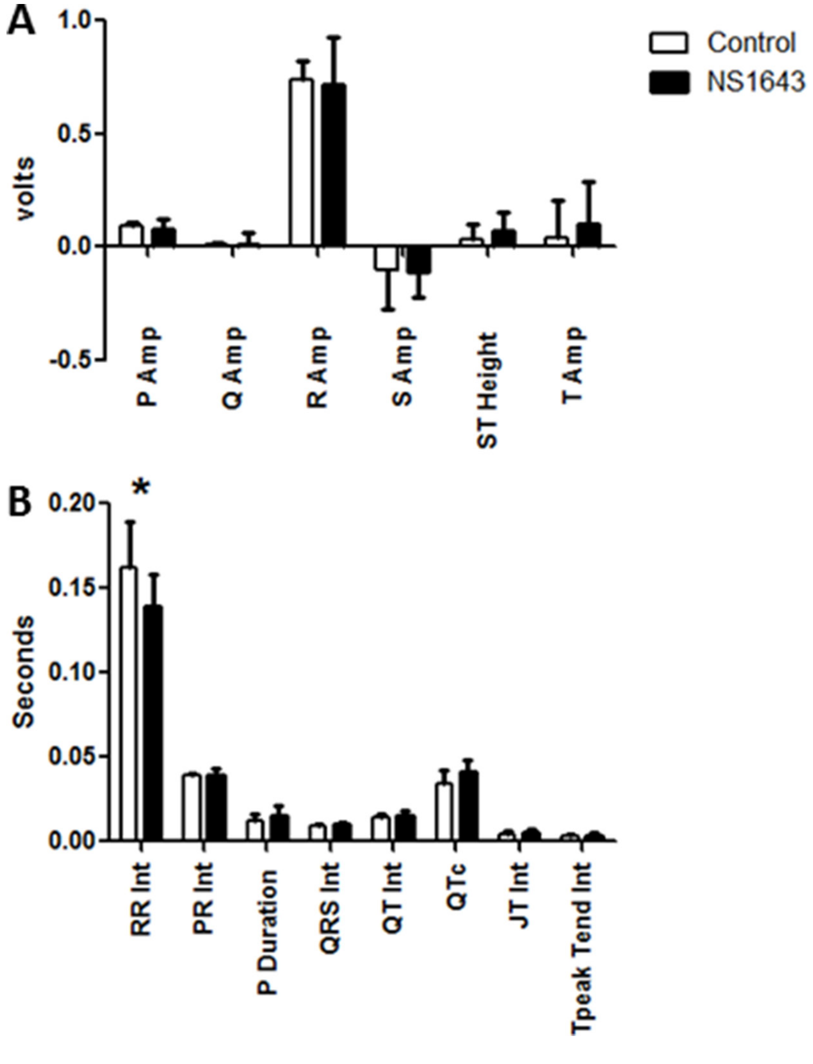

Supplementary Figure 4: Effect of NS1643 on the mice heart electrical activity. Analyses of the effect of NS1643 on the QRST (LabChart8; ADInstruments) complex of the mice's heart treated with NS1643. An increase in heart rate in NS1643 treated mice is attributed to a decrease in R-R interval. Int = interval; Amp = amplitude. $^{*}p < 0.05$.
